# Eco-Friendly Production of Lignin-Containing Cellulose Nanofibers from Sugarcane Bagasse Fines via Sequential Thermal Hydrolysis–Deep Eutectic Solvents Pretreatment

**DOI:** 10.3390/polym18010085

**Published:** 2025-12-27

**Authors:** Chae-Eun Yeo, Ho-Jin Sung

**Affiliations:** Energy and Environment Research Center, Institute for Advanced Engineering, Yongin 17180, Republic of Korea; yce@iae.re.kr

**Keywords:** sugarcane bagasse, lignin-containing cellulose nanofibers, microfluidization, particle size, unutilized biomass

## Abstract

Fine and ultra-fine sugarcane bagasse (SCB) fractions (≤200 μm) that are naturally generated during industrial grinding have been systematically overlooked in lignocellulosic pretreatment research. Previous studies have largely relied on commercially processed pulps or coarse particles (>200 μm), typically without systematic size fractionation. Here, we demonstrate that these fine fractions—including ultra-fines (≤45 μm), which are often excluded from analytical workflows due to concern about excessive degradation—are viable feedstocks for producing lignin-containing cellulose nanofibers (LCNF) via a sequential thermal hydrolysis treatment (THT)–deep eutectic solvent (DES) pretreatment specifically designed to retain lignin. Size-fractionated SCB (≤45, 45–100, and 100–200 μm) was subjected to THT (190 °C, 15 min), followed by DES treatment using choline chloride/urea (1:2 molar ratio, 130 °C, 2 h). Multi-technique characterization using Fourier transform infrared spectroscopy (FT-IR), thermogravimetric analysis (TGA), and X-ray diffraction (XRD) indicated substantial hemicellulose removal (>70%), effective lignin retention (7.6–9.1%), cellulose enrichment (74.0–77.5%), and preservation of cellulose I structure allomorph. The crystallinity index increased from 46.5–52.7% after THT to 56.7–57.2% after DES treatment, and notably, uniform compositional and structural features were obtained across all particle size classes after DES treatment. Subsequent high-pressure microfluidization (700 bar, five passes) yielded LCNF with consistent morphology across all fractions: uniform fibril diameters (24.6–26.2 nm), a discernible lignin coating, and excellent colloidal stability (zeta potential: −86.3 to −95.0 mV). Field emission scanning electron microscopy (FE-SEM) and transmission electron microscopy (TEM) confirmed well-dispersed nanofibrous networks. Collectively, these findings show that the full range of fine SCB fractions can be effectively valorized into high-performance LCNF through sequential THT–DES pretreatment, enabling comprehensive utilization of industrial grinding outputs and advancing circular bioeconomy objectives.

## 1. Introduction

Sugarcane bagasse (SCB) is a major agro-industrial residue generated in substantial quantities by the sugar and ethanol industries [1,2]. Globally, approximately 540 million tons of SCB are produced each year [3], rendering it an abundant, low-cost, and readily accessible lignocellulosic resource [4]. As a typical lignocellulosic biomass, SCB consists of roughly 40–50% cellulose, 20–35% hemicellulose, and 18–24% lignin on a dry weight basis [1,5,6]. This compositional richness positions SCB as a promising feedstock for conversion into a range of value-added products, including biofuels, biomaterials, and adsorbents [7].

Despite this potential, SCB remains underutilized [4]. Approximately 50% of SCB was combusted as boiler fuel to meet the energy demands of sugar mills [1,3,7], leading to substantial surplus that is frequently discarded [1,4]. The valorization of this residual bagasse into high-value materials has therefore become an urgent priority, and it has attracted increasing attention within biorefinery frameworks in response to growing environmental concerns [8,9,10,11].

One promising strategy is the conversion of SCB into nanocellulose, commonly referred to as cellulose nanofibers (CNF) [2]. Nanocellulose is an emerging material characterized by low cost, biocompatibility, biodegradability, and versatile surface chemistry, enabling high-value addition and broad applicability across industries [12,13]. Accordingly, producing nanocellulose from SCB represents a waste-to-wealth approach that maximizes the sustainable utilization of biomass resources.

Lignin-containing cellulose nanofibers (LCNF) offer additional advantages by eliminating or substantially reducing the bleaching and complete delignification steps required in conventional cellulose nanomaterial isolation. This simplification decreases process complexity, energy demand, chemical consumption, and waste generation [14,15]. Consequently, LCNF production can lower both cost and environmental impact [14], making it a promising platform for generating high-value products from low-grade or underutilized agro-industrial biomass [16].

Moreover, residual lignin can impart functionality to LCNF, including hydrophobicity (an intrinsic property of lignin), strong UV-blocking capacity, and antioxidant and antimicrobial activities associated with lignin’s aromatic phenolic structures [14,17]. These features broaden the application potential of LCNFs in high-performance composites [18,19], environmentally friendly packaging and barrier materials [20], flexible electronics [21,22], energy storage systems [23], and robotic flexible protective layers [24]. Such applications leverage the mechanical robustness, barrier performances, thermal stability, and lignin-derived electrochemical functionality of LCNF. For instance, high stiffness coupled with low density benefit the composites’ reinforcement, whereas enhanced barrier properties and optical transparency are advantageous for packaging. Overall, the combined mechanical strength, thermal stability, and lignin-derived functionality position LCNF as a next-generation nanomaterial for sustainable industrial applications.

The primary barrier to the commercialization of CNF production is the high energy demand associated with high-intensity shear processes required for nanofiber defibrillation [25]. Although chemical pretreatments are widely used to mitigate this limitation, conventional strong acid/alkali treatments often struggle to satisfy commercialization and environmental requirements because of toxicity, high costs, and time-consuming procedures [26].

Previous SCB-based LCNF production routes have pursued lignin retention through various strategies, including soda-oxygen pulping (4.5–16.1% lignin; harsh alkaline conditions) [27], organosolv-TEMPO oxidation (5–7.5% lignin; toxic NaClO reagents) [28], steam explosion coupled with enzymatic hydrolysis (4.8–11.6% lignin; high energy input) [29], and hydrothermal-maleic acid treatment (17–26% lignin; corrosive acids) [30]. In general, these approaches require energy-intensive processing, harsh chemical reagents, or both, which constrains the environmental and economic feasibility of sustainable LCNF production.

Deep eutectic solvents (DES), particularly choline chloride-based systems, have emerged as greener alternatives for biomass fractionation owing to their low toxicity, biodegradability, and recyclability [31]. ChCl:urea can selectively cleave lignin β-O-4 ether linkages—the predominant interunit bond in native lignin—while preserving the cellulose I structure, thereby facilitating subsequent mechanical defibrillation [32,33]. However, single-step DES treatment can lead to either incomplete hemicellulose removal under mild conditions or excessive delignification under harsh conditions, limiting control over the final lignin content that is critical for LCNF functionality.

THT primarily promotes hemicellulose removal via autohydrolysis, in which acetyl groups in hemicellulose generate organic acids under elevated temperature and pressure, thereby catalyzing polysaccharide hydrolysis through acid-catalyzed cleavage of glycosidic bonds [34,35]. Subsequently, DES enables selective lignin modification and partial extraction by cleaving β-O-4 ether linkages and disrupting the lignin-carbohydrate complex, while maintaining the cellulose I crystal structure [36,37,38]. This sequential strategy is consistent with green chemistry principles [39] because it employs bio-based solvents, minimize chemical inputs, and operates under relatively mild conditions, thereby avoiding the toxic effluents and high energy demands associated with conventional alkaline/oxidative delignification.

However, industrial-scale grinding of lignocellulosic biomass such as SCB inevitably yields broad particle size distributions that contain substantial amounts of fines (typically <50 μm), rather than narrow and uniform size fractions [40]. Particle size directly affects pretreatment performance by governing reagent diffusion, available reaction surface area, and mass transfer rates, making it a critical factor determining pretreatment efficiency and compositional changes, and thus represents a key determinant of compositional changes and final nanofiber quality [41]. Nevertheless, most prior studies have assessed pretreatment and saccharification using relatively coarse particles (0.5–4 mm) [30,38,39,42,43]. Moreover, NREL compositional analysis protocols are explicitly optimized for knife-milled samples above 0.18 mm, where fines can cause analytical complications such as pseudo-lignin formation [43,44,45].

This gap is particularly important because fine fractions can exhibit distinct compositional characteristics, including a disproportionately high inorganic content in herbaceous feedstocks [43,45]. As summarized in Table 1, recent DES-based pretreatment studies on lignocellulosic biomass have largely overlooked fine particle fractions (<200 μm)—especially fines (<100 μm)—despite their prevalence in industrial grinding outputs (30–40% of total mass [33]).

Among the surveyed studies (Table 1), none performed systematic particle size fractionation or conducted comparative analysis across size classes, highlighting a critical knowledge gap. Most studies relied on commercially processed pulps or “as-received” biomass—often without reporting particle size distributions, or by focusing only on coarse fractions > 200 μm)—and therefore did not assess the feasibility of sub-millimeter fractions. Notably, pyrolysis kinetics studies that examine individual lignocellulosic components commonly employ fines as small as 50–100 µm to minimize internal heat- and mass transfer limitations, implying high reactivity at these particle dimensions [52]. In contrast, biomass pretreatment studies frequently neglect or discard fines due to concerns regarding reduced solid recovery caused by excessive degradation [53,54]. This practice can lead to missed valorization opportunities, despite the potential advantages of fines in improving pretreatment efficiency.

Furthermore, all surveyed studies applied single-step DES pretreatment primarily aimed at delignification, whereas the present study investigates a sequential THT–DES pretreatment specifically tailored for LCNF production with lignin retention.

Beyond the particle size knowledge gap identified in Table 1, preserving lignin during LCNF production remains a central challenge across diverse lignocellulosic feedstocks (Table 2). This study addresses three key gaps that have not been systematically examined in prior SCB-based LCNF research: (i) comprehensive characterization of compositional and structural properties across representative sub-millimeter fractions (≤45, 45–100, and 100–200 μm); (ii) development of sequential THT-DES pretreatment strategies designed for lignin retention, in contrast to conventional single-step DES delignification approaches that yield lignin-free substrates; and (iii) experimental validation that all evaluated sub-millimeter fractions—including fines (≤45 μm), which are often discarded in conventional processing—can serve as viable precursors for LCNF, thereby enabling full valorization of industrial SCB grinding outputs.

For LCNF-oriented processes, precursor pulp quality (i.e., cellulose enrichment with appropriately controlled lignin removal) and the energy efficiency of the pretreatment–mechanical nanofibrillation sequence are more relevant metrics than final sugar yield. From this, strategies that utilize the full particle size distribution represent an unexplored opportunity. In general, reducing particle size increases specific surface area and enhances mass transfer, which can improve pretreatment performance and facilitate subsequent mechanical nanofibrillation. However, finer particles may also exacerbate solid loss and degradation, reducing solid recovery [53,54]. Despite these considerations, comprehensive studies assessing whether such small-size fractions—particularly those typically discarded—can be converted into viable LCNF precursors after mild THT–DES pretreatment remain scarce. If validated, these fractions could enable valorization of the entire industrial grinding output, maximizing resource efficiency and supporting circular bioeconomy principles.

Accordingly, the primary objective of this work is to demonstrate that sub-millimeter SCB particles, including the ultra-fine fraction, that can serve as effective LCNF precursors under environmentally benign conditions. This approach offers three practical advantages: (1) effective lignin preservation; (2) environmentally benign processing (mild operating conditions, a recyclable solvent, and avoidance of harsh chemicals); and (3) applicability across all sub-millimeter fractions.

Specifically, this study pursues key objectives: (i) to experimentally demonstrate that the combined THT–DES pretreatment constitutes an environmentally benign, potentially DES-recyclable, and scalable pathway for LCNF production, thereby avoiding the toxicity and high energy demands of conventional strong acid/alkali processes; (ii) to systematically characterize the compositional and structural properties of three representative sub-millimeter SCB fractions (<45, 45–100, and 100–200 μm) after THT–DES pretreatment using chemical composition analysis, Fourier transform infrared spectroscopy (FT-IR), X-ray diffraction (XRD), and thermogravimetric analysis (TGA), thereby addressing the gap highlighted in Table 1; (iii) to demonstrate the practical viability of all fractions as LCNF precursors by evaluating nanofiber morphology, diameter distribution, and dispersion behavior using dynamic light scattering (DLS) and transmission electron microscopy (TEM) analysis; and (iv) to provide fundamental data to guide the design of LCNF production processes that rationally utilize the full particle size distribution from industrial SCB grinding, rather than discarding fine fractions—thereby maximizing biomass valorization and advancing a circular bioeconomy framework.

## 2. Materials and Methods

### 2.1. Materials

SCB was obtained from a local supplier in Korea. Urea (≥99%) and choline chloride (ChCl, ≥99%) used for preparing the deep eutectic solvent (DES) preparation were purchased from Samchun Pure Chemical Co., Ltd. (Seoul, Republic of Korea). All other chemicals were of analytical grade and used as received without further purification. Deionized water (18.2 MΩ·cm) was used throughout all experiments.

### 2.2. Size Fractionation

Size fractionation was performed to evaluated the effects of particle size on pretreatment efficiency. Air-dried SCB was first milled using a hammer/cutter mill (POLYMIX^®^, PX-MFC 90D, Kinematica AG, Lucerne, Switzerland) and subsequently separated into three particle size ranges (≤45, 45–100, and 100–200 μm) using standard test sieves (Daehan Scientific Co., Ltd., Seoul, Republic of Korea) compliant with ASTM E11-24 [56] and ISO 3310-2:2013 [57] (mesh Nos. 325, 140, and 80, respectively). Biomass particles were fractionated following established sieve-based protocols commonly used in lignocellulosic biomass research [22]. Cross-contamination between fractions was minimized by thorough sieve cleaning and blank runs between fractionation steps. The fractionated samples were sealed in polyethylene bags and stored at ambient temperature until further use.

#### Sample Nomenclature

Throughout this study, samples are designated using the format [Pretreatment]-[Fraction], where the fraction label corresponds to the nominal sieve cut size (upper bound) or the size range:

- F45: <45 μm (ultra-fine fraction)
- F100: 45–100 μm (fine faction)
- F200: 100–200 μm (medium-fine fraction)

### 2.3. Preparation of Lignin-Containing Cellulose Nanofibers (LCNF)

**Figure 1:**
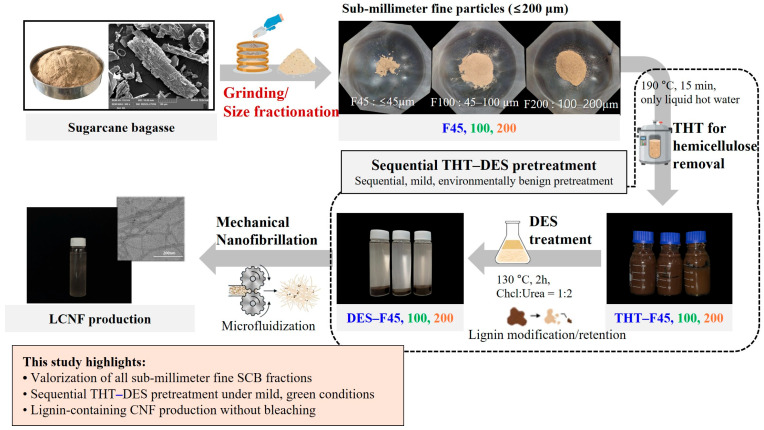
Schematic illustration of LCNF production from sub-millimeter fine SCB fractions via sequential THT–DES pretreatment and mechanical nanofibrillation.

A schematic overview of the overall process for producing LCNF from underutilized SCB is presented in Figure 1. Briefly, air-dried SCB was first milled and sieved into three particle size fractions (F45, F100, and F200) as described in Section 2.2. Each fraction was then subjected to sequential thermal hydrolysis and DES pretreatment (Section 2.3.1 and Section 2.3.2), followed by mechanical nanofibrillation via high-pressure microfluidization (Section 2.3.3) to obtain LCNF.

#### 2.3.1. Thermal Hydrolysis Treatment (THT)

THT was conducted for size-fractionated SCB samples in stainless-steel autoclaves using deionized water at a solid-to-liquid ratio of 1:20 (*w*/*v*). The reaction was performed at 190 °C for 15 min under autogenous pressure (approximately 12.5 bar, generated by saturated steam in the closed system), followed by cooling to room temperature. The resulting slurry was washed with deionized water and vacuum-filtered to remove dissolved non-cellulosic components.

#### 2.3.2. DES Preparation and Treatment

The DES was prepared by mixing choline chloride and urea at a molar ratio of 1:2 and heating at 80 °C until a homogeneous, clear liquid formed. DES treatment was performed in sealed vessels at 130 °C for 2 h under mechanical stirring [58]. After treatment, the suspension was separated into solid and supernatant phases by filtration. The solid fraction was washed repeatedly with deionized water until the supernatant reached neutral pH (≈7) [46].

#### 2.3.3. Mechanical Defibrillation (Microfluidization)

Following pretreatment, the solid fraction was recovered by vacuum filtration and washed with deionized water until the pH of the washing remained constant. The resulting suspension was adjusted to 1 wt% and then processed using a high-shear fluid processor (M110P, Microfluidics Corp., Westwood, MA, USA) at 700 bar for 5 passes. The processing temperature was maintained below 50 °C. The obtained LCNF suspension were stored at 4 °C until further characterization.

### 2.4. Characterization of Cellulose and LCNF

#### 2.4.1. Chemical Composition Analysis

The chemical composition (cellulose, hemicellulose, and lignin) was determined according to NREL/TP-510-42618 protocols [42]. Thermogravimetric analysis (TGA, Section 2.4.4) and FT-IR spectroscopy (Section 2.4.2) were used as complementary validation techniques. Component assignments were based on sequential acid hydrolysis and further corroborated by characteristic thermal decomposition behavior [42] and spectroscopic signatures. Results are reported as mean ± standard deviation (*n* = 3) and agreement across analytical methods was used to confirm data reliability.

#### 2.4.2. Fourier Transform Infrared Spectroscopy (FTIR)

FTIR spectra were collected using a Nicolet iS50 FT-IR spectrometer (Thermo Fisher Scientific, Waltham, MA, USA). Dried samples were analyzed at room temperature over a wavenumber range of 4000–400 cm^−1^ with a spectral resolution of 4 cm^−1^ and 32 co-added scans. Data acquisition and processing were performed using OMNIC software. For clarity, spectra were vertically offset for visualization.

#### 2.4.3. X-Ray Diffraction Analysis (XRD)

The crystal structure of the samples was examined using an X-ray diffractometer (XRD, SmartLab SE, Rigaku Corp., Tokyo, Japan). Dried powders were loaded into a glass sample holder and gently leveled to obtained a flat surface. Measurements were conducted using Cu-Kα radiation (λ = 1.5418 Å) at 40 kV and 30 mA. Diffraction patterns were recorded over a 2θ range of 10–80° at a scan rate of 5°/min. The crystallinity index (*CrI*) of cellulose was calculated using the Segal method [59] according to the following equation:

(1) $$CrI\;\left(\%\right)=\;\left[\left(I_{200}-I_{am}\right)\;/I_{200}\right]\times 100$$

where $I_{200}$ is the maximum diffraction intensity of the reflection associated with the crystalline region near 2θ = 22.5°, and $I_{am}$ is the minimum intensity corresponding to the amorphous region near 2θ = 18°. All diffraction patterns were normalized to the most intense peak (100%) and vertically offset for clarity.

#### 2.4.4. Thermal Gravimetric Analysis (TGA)

Thermal stability and decomposition behavior were evaluated by thermogravimetric analysis (TGA) using a simultaneous thermal analyzer (STA 449 F5 Jupiter^®^ Eco-Line, NETZSCH, Gerätebau GmbH, Selb, Germany). Prior to analysis, samples were dried and ground into fine powder using a mortar and pestle. Approximately 10 mg of dried powder was placed in a platinum pan and heated from room temperature to 800 °C at a constant heating rate of 10 °C/min under a nitrogen atmosphere (20 mL/min) to minimize oxidative degradation.

#### 2.4.5. Zeta Potential Measurement

The colloidal stability of LCNF dispersions was assessed by measuring zeta potential using a dynamic light scattering (DLS) instrument (NANO-flex II, Colloid Metrix GmbH, Meerbusch, Germany) at a sample concentration of 0.1 wt%. Prior to measurement, samples were dispersed by gentle stirring and ultrasonication to disrupt aggregates, and measurements were conducted immediately after dispersion. Each sample was analyzed in quadruplicate, and results are reported as mean ± standard deviation (*n* = 4).

#### 2.4.6. Field Emission Scanning Electron Microscopy (FE-SEM) and Transmission Electron Microscopy (TEM)

Surface morphology was examined using a field emission scanning electron microscope (FE-SEM, Mira 3, Tescan Orsay Holding a.s., Brno, Czech Republic). Dried samples were mounted on aluminum stubs using conductive carbon tape and sputter-coated with a gold layer (~10–15 nm) under vacuum. Images were acquired at an acceleration voltage of 5 kV.

The morphology of lignin-containing cellulose nanofibers (LCNF) was further characterized using a transmission electron microscope (TEM, Talos F200X, Thermo Fisher Scientific Inc., USA) operated at 200 kV. TEM specimens were prepared by diluting the LCNF suspension with distilled water; a drop of the diluted suspension was deposited onto a carbon-coated copper grid (200 mesh) and dried at room temperature.

Quantitative fibril diameter analysis was performed using ImageJ v1.54 software (National Institutes of Health, Bethesda, MD, USA) [60] on both FE-SEM and TEM micrographs. For each sample, at least 50 well-defined nanofibers were measured from regions where individual fibrils were clearly distinguishable to ensure statistical reliability.

## 3. Results and Discussions

### 3.1. Chemical and Compositional Characterization

**Figure 2:**
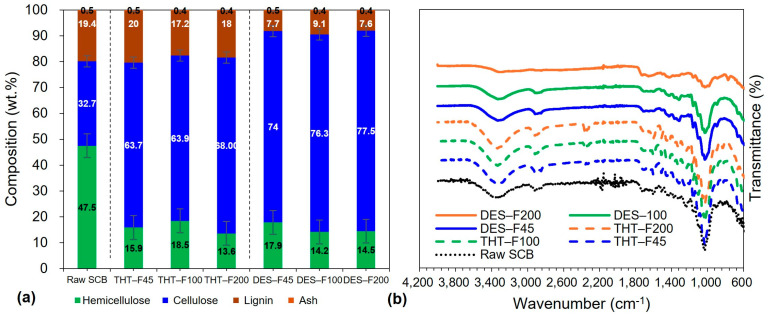
Chemical and compositional characterization of raw SCB and sequentially pretreated samples: (**a**) Chemical composition demonstrating progressive cellulose enrichment, hemicellulose removal, and partial lignin retention; (**b**) FT-IR spectra showing stepwise attenuation of hemicellulose and lignin-associated bands with preservation of the cellulose backbone.

Figure 2 summarizes the chemical and compositional characterization of raw SCB and sequentially pretreated samples. Figure 2a shows the compositional changes during sequential pretreatment. Raw SCB contained 47.5% hemicellulose, 32.7% cellulose, 19.4% lignin, and 0.5% ash. After the first stage (THT), hemicellulose decreased to 15.9–18.5% (65–67% removal), while cellulose increased to 63.7–64.0% and lignin remained relatively stable at 17.2–20.0%. Following the second stage (DES), hemicellulose was further reduced to 14.2–17.9% (overall > 70% removal from raw SCB), cellulose increased to 74.0–77.5%, and lignin decreased to 7.6–9.1%.

Notably, the retained lignin content (7.6–9.1%) in DES-treated samples indicates that the sequential THT–DES pretreatment yields suitable precursors for LCNF production, since LCNF typically requires >1% lignin to retain hydrophobic and antioxidant functionalities distinct from lignin-free CNF [61]. These values are consistent with reported ranges for DES-pretreated substrates (cellulose: 70–80%) and with typical LCNF specifications (lignin: 5–15%), supporting the effectiveness of the sequential pretreatment [61,62]. This compositional profile—high cellulose enrichment coupled with substantial functional lignin retention—distinguishes the present mild sequential pretreatment from harsh delignification routes that produce lignin-free substrates (<1% lignin). The retained lignin is expected to underpin key LCNF functionalities, including enhanced hydrophobicity, UV-blocking performance, and antioxidant properties [63]. Importantly, DES-treated samples exhibited relatively uniform composition across particle size fractions: cellulose ranged from 74.0% (DES-F45) to 77.5% (DES-F200), a difference of 3.5% percentage points, while lignin ranged from 7.7% (DES-F45) to 9.1% (DES-F100), indicating compositional homogeneity that is advantageous for consistent LCNF production.

The compositional trends were further supported by FT-IR spectroscopy (Figure 2b), which compares raw SCB and samples after the first stage (THT-F45, THT-F100, THT-F200) and second stage (DES-F45, DES-F100, DES-F200). All spectra exhibited typical lignocellulosic features, including broad O–H stretching near 3400 cm^−1^ (cellulose, hemicellulose, lignin, and absorbed moisture) [40] and C–H stretching near 2900 cm^−1^ associated with aliphatic structures [41]. The intensity of the ~2900 cm^−1^ band increased progressively from raw SCB to THT-treated and then to DES-treated samples, consistent with the marked cellulose enrichment from 32.7% to 74.0–77.5% (Figure 2a) [64].

The carbonyl (C=O) band at ~1730 cm^−1^, attributed to acetyl and ester groups in hemicellulose [38], decreased markedly after the first stage and was nearly absent after the second stage (Figure 2b). This spectroscopic change directly corroborates the progressive hemicellulose removal observed in the compositional analysis (47.5%→14.2–17.9%; Figure 2a). Consistent with the expected mechanisms, THT primarily depolymerizes hemicellulose via autohydrolysis, whereas DES further removes residual hemicellulosic fragments through the disruption of hydrogen bonding interactions. The extent of reduction is in agreement with reported efficiencies of 60–80% for THT alone and >90% for combined strategies [62]. Efficient hemicellulose removal is critical for LCNF production because it increases cellulose accessibility, exposes cellulose microfibrils, and thereby improves the efficiency of subsequent mechanical fibrillation [65].

Lignin-associated bands at 1600–1510 cm^−1^ (aromatic C=C stretching) and ~1460 cm^−1^ (C–H deformation) [43] exhibited a similar stepwise attenuation. These features were prominent in raw SCB, decreased moderately after THT, and declined substantially after DES treatment. Importantly, aromatic signals remained clearly detectable in all DES-treated samples, consistent with the compositional results indicating 7.6–9.1% lignin retention (Figure 2a). This spectral persistence differentiates the present mild sequential pretreatment from harsh delignification approaches that remove lignin to the extent that aromatic bands are no longer observed [61]. The maintained aromatic signatures at 1600–1510 cm^−1^ further suggest that the retained lignin remains structurally intact, supporting its functional contributions to LCNF properties such as hydrophobicity and antioxidant capacity [63,66].

The crystallinity-sensitive ratio A_1430_/A_898_, assigned to crystalline-to-amorphous cellulose bands [2], increased progressively from raw SCB to THT and then to DES-treated samples, indicating stepwise removal of amorphous hemicellulose and partial removal of amorphous lignin. This trend is consistent with the observed compositional shifts toward higher cellulose content (Figure 2a). In addition, the cellulose backbone band near 1050 cm^−1^ (C–O–C vibrations of the pyranose ring) remained prominent across all samples, indicating preservation of cellulose chain integrity, which is essential for achieving high mechanical performance in LCNF [67].

After completion of sequential pretreatment, particle sizes dependent on different composition and spectral features were minimal. DES-treated fractions displayed high similar FT-IR profiles and narrow compositional ranges (cellulose: 74.0–77.5%, lignin: 7.6–9.1%) regardless of initial particle size. This convergence is plausibly attributable to effective DES penetration into fiber structures that were preconditioned by THT, thereby diminishing initial size-related disparities. Collectively, the compositional and spectroscopic uniformity—together with preserved cellulose structure (1050 cm^−1^), retained lignin signatures (aromatic bands), and increased A_1430_/A_898_—indicates chemically homogeneous substrates across the fine particle size range. Such homogeneity is crucial for producing LCNF with consistent and predictable properties. Moreover, the >70% hemicellulose removal and preserved cellulose integrity are expected to facilitate efficient mechanical nanofibrillation, while the retained lignin content (7.6–9.1%) supports LCNF classification and associated functional advantages required in applications where hydrophobicity and antioxidant performance are beneficial.

**Figure 3:**
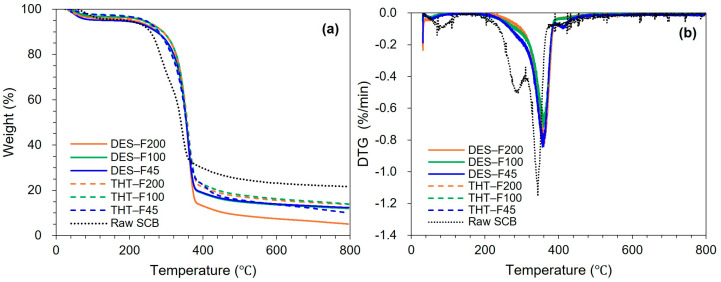
TGA curves of raw SCB and sequentially pretreated samples: (**a**) TGA curves showing weight loss profiles and (**b**) DTG curves showing the degradation rates as a function of temperature.

Figure 3 shows the TGA and derivative thermogravimetric (DTG) curves of raw SCB and sequentially pretreated samples. All samples exhibited the characteristic three-stage thermal degradation behavior of lignocellulosic bioweight: (i) moisture loss at 30–120 °C, (ii) major devolatilization/decomposition at 200–400 °C, and (iii) slow degradation of the residual char above 400 °C [44].

Raw SCB exhibited a pronounced DTG maximum at ~340 °C and a distinct low-temperature shoulder at 220–280 °C, which is typically associated with hemicellulose decomposition (Figure 3b). This concentrated degradation behavior reflects the overlapping thermal decomposition of hemicellulose (220–315 °C), cellulose (315–400 °C), and lignin (160–900 °C) within the intact lignocellulosic matrix. In addition, synergistic effects can intensify weight loss, as acidic byproducts generated during hemicellulose degradation may accelerate cellulose depolymerization [68,69]. After the first stage pretreatment (THT), the low-temperature shoulder was nearly eliminated, consistent with effective hemicellulose removal via autohydrolysis. Concurrently, the main DTG peak shifted to 350–360 °C and become broader with reduced intensity (−0.7–0.8%/min), indicating removal of thermally labile components and mitigation of synergistic degradation (Figure 3b). Notably, THT-treated samples (THT-F45, THT-F100, and THT-F200) displayed comparable thermal behavior across particle size fractions, with onset temperatures of 285–290 °C and substantially reduced char residues at 800 °C. These trends are consistent with hemicellulose removal and cellulose enrichment observed in compositional and FT-IR analysis.

Following the second-stage DES treatment, the DTG profiles broadened further, and the main peak shifted to higher (355–365 °C) with slightly lower peak intensity (−0.7–0.75%/min). This behavior is consistent with the progressive enrichment of more crystalline cellulose, which decomposes more gradually and at higher temperatures [69,70]. In addition, DES-treated samples exhibited enhanced weight loss in the 400–600 °C region, which is characteristic of residual lignin degradation, while producing less char at 800 °C than the THT-treated samples. These observations indicate further removal of thermally stable lignin and inorganic components during the DES stage, in agreement with the attenuated aromatic features in FT-IR spectra and previous reports on DES-mediated delignification [71].

Notably, DES-treated samples displayed minimal particle size-dependent variation in the main decomposition temperature, with peak temperatures differing by less than 5 °C and onset temperatures of 305–310 °C—approximately 20 °C higher than those of the THT samples. This thermal convergence suggests a high degree of compositional homogeneity, which is important for producing LCNF with consistent properties. The low char yield across all DES-treated fractions further indicates near-complete removal of thermally labile non-cellulosic components, resulting in substrates with high cellulose purity that are well suited for subsequent mechanical nanofibrillation. Collectively, these thermal features—preservation of the cellulose decomposition maximum (~360 °C), reduced char formation, and uniform degradation profiles—support the suitability of the DES-treated samples as thermally stable and chemically consistent LCNF precursors across the fine particle size range.

**Figure 4:**
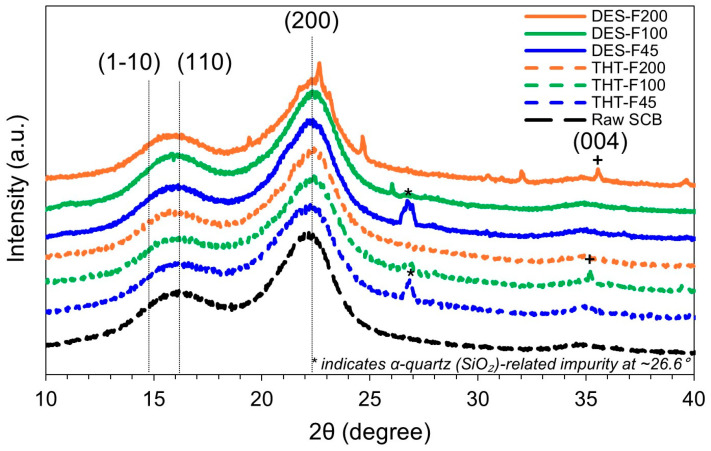
XRD patterns of raw SCB and samples after sequential THT–DES pretreatment. Characteristic cellulose I reflections are assigned to $2\theta \approx 14.8{}^{\circ}\;\left(1\bar{1}0\right),\;16.4{}^{\circ}\;\left(110\right),\;and\;22.5{}^{\circ}\;\left(200\right)$. A weak cellulose I (004) reflection near 2θ≈34.5–35.0° is observed in selected samples (DES-F200 and THT-F100). The sharp peak at 2θ≈26.6° (*) is attributed to residual silica (α-quartz) impurities.

Figure 4 shows the XRD patterns of raw SCB and sequentially pretreated samples collected over the 2θ range of 10–40°. Samples after the first stage (THT-F45, THT-F100, and THT-F200) and after the second stage (DES-F45, DES-F100, and DES-F200) exhibited distinct diffraction peaks characteristic of cellulose I structure at $2\theta \approx 14.8{}^{\circ}\;(1\bar{1}0)$ and 2θ≈16.4°(110), 22.5°(200). These reflections confirm that the native cellulose Iβ lattice was preserved throughout the process, with no detectable conversion to cellulose II or other polymorphs [23,43].

In addition to the primary cellulose I reflections, secondary features were observed in specific fractions. A sharp peak at 2θ≈26.6°, observed in THT-F45 and DES-F45, is consistent with α-quartz. detected in THT-F45 and DES-F45, is consistent with α-quartz, and is likely attributable to the silica-rich inorganic residues (e.g., plant- or soil-derived ash) associated with sugarcane bagasse [72]. Moreover, a weak reflection assigned to the cellulose I (004) plane was observed at 2θ≈34.5−35.0° in DES-F200 and THT-F100, suggesting enhanced longitudinal ordering along the fiber axis [73]. Importantly, because these minor reflections are well-separated from the (200) peak and from the minimum intensity near ~18° (I_am_; amorphous contribution) used in the Segal method, they do not materially affect peak assignment or *CrI* estimation [69].

*CrI* values calculated using the Segal method [59] showed particle size-dependent trends after THT (46.49–52.66%), as summarized in Table 3. The finer fractions (THT-F45) exhibited a slightly lower *CrI*, which may reflect greater structural disruption during thermomechanical processing, whereas the coarser fraction (THT-F200) showed a higher *CrI*, consistent with the preferential removal of amorphous hemicellulose. After DES treatment, *CrI* increased uniformly to across all fractions to 56.69–57.19%. These values agree with the literature ranges reported for comparable stages: untreated lignocellulosic biomass typically exhibits *CrI* of 45–50% [74], THT yields intermediate *CrI* values of 45–55% depending on the extent of hemicellulose removal [75], and mild chemical pretreatments for CNF production commonly result in final *CrI* values of 55–65% [76].

Within the THT samples, *CrI* varied by 6.17% percentage points across particle sizes (46.49–52.66%), reflecting the combined effects of hemicellulose removal and structural disruption. In contrast, the DES stage produced consistently high crystallinity (56.69–57.19%, difference of <0.50% percentage points), indicating effective removal of residual amorphous components irrespective of initial particle size. The convergence to a literature-consistent final *CrI* (~57%) further supports that the sequential THT–DES pretreatment generates cellulose-enriched substrates with preserved native structure, making them suitable precursors for mechanical nanofibrillation into LCNF.

**Figure 5:**
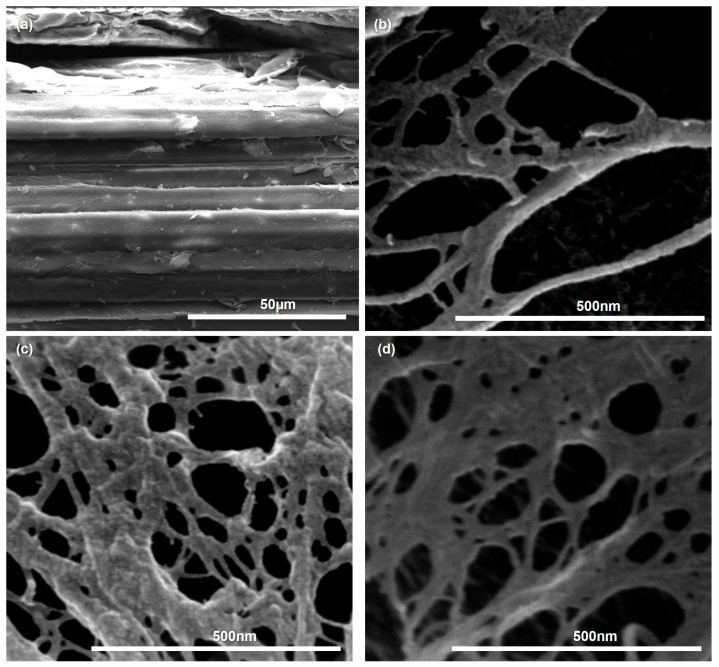
FE-SEM images of (**a**) raw SCB and (**b**–**d**) DES-treated samples after sequential THT–DES pretreatment: (**b**) DES-F45 (≤45 μm fraction), (**c**) DES-F100 (45–100 μm fraction), and (**d**) DES-F200 (100–200 μm fraction).

FE-SEM micrographs (Figure 5) illustrate the morphological changes induced by the sequential THT–DES pretreatment across all particle size fractions. Raw SCB (Figure 5a) exhibited intact, densely packed fiber bundles with layered features typical of untreated lignocellulosic biomass. After THT–DES treatment, all fractions (DES-F45, DES-F100, and DES-F200; Figure 5b–d) showed loosened and partially unwound fiber bundles and partially delaminated layered structures, resulting in a mixed morphology comprising porous, sheet-like networks interspersed with fine fiber bundles. Notably, no substantial morphological differences were observed among the three DES-treated fractions, indicating that the sequential pretreatment effectively weakened interfibrillar bonding across the entire fine particle size range (≤200 μm). These observations are consistent with previous studies showing that mild chemical pretreatment can promote fiber delamination without complete delignification [61]. Such structural modification is expected to yield precursor substrates that are amenable to efficient mechanical nanofibrillation into LCNF.

### 3.2. Morphological and Colloidal Properties of LCNF

**Figure 6:**
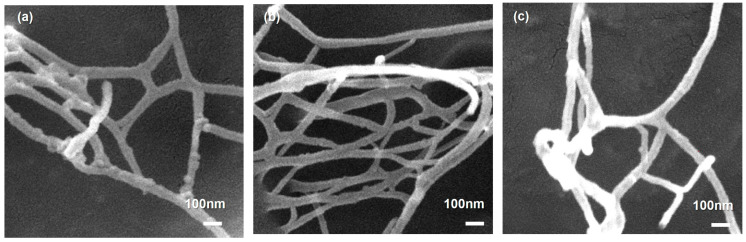
FE-SEM image of dried LCNF samples: (**a**) LCNF-F45, (**b**) LCNF-F100, and (**c**) LCNF-F200. All micrographs were acquired at ×50,000 magnification.

The morphology of LCNF produced by sequential THT–DES pretreatment followed by mechanical nanofibrillation was examined by FE-SEM and TEM. FE-SEM images (Figure 6) revealed highly entangled, three-dimensional nanofibrillar networks for fractions (LCNF-F45, LCNF-F100, and LCNF-F200), with minimal aggregation, indicating effective mechanical defibrillation. The three samples exhibited comparable network morphologies with no discernible fraction-dependent differences, although occasional localized variations in fibril density and bundling occurred. These local variations are attributable to spatial heterogeneity during sample preparation and drying rather than intrinsic differences among the materials. The resulting net structure—characterized by high-aspect-ratio fibrils and extensive interfibrillar entanglement—is typical of mechanically produced LCNF and is advantageous for mechanical reinforcement applications [61,77].

**Figure 7:**
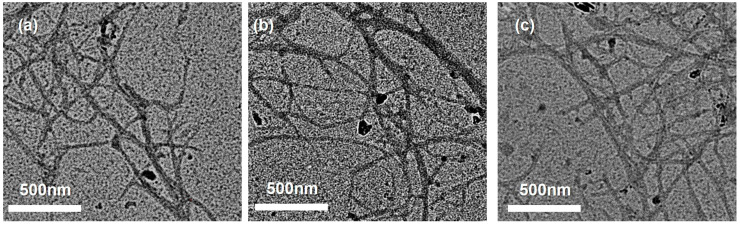
TEM images of LCNF samples: (**a**) LCNF-F45, (**b**) LCNF-F100, and (**c**) LCNF-F200. Individual nanofibrils exhibit diameters of 10–50 nm and lengths of several micrometers. Darker contrast along fibril surfaces suggests a retained lignin coating, consistent with the compositional analysis (7.6–9.1% lignin; Figure 2a).

TEM imaging (Figure 7) provided complementary high-resolution visualization of individual LCNF across all size fractions. Well-dispersed nanofibrils extending several micrometers in length were observed, confirming effective mechanical defibrillation and liberation of cellulose microfibrils from the lignocellulosic matrix. Notably, darker contrast along fibril surfaces—particularly apparent in Figure 7—suggests the presence of a residual lignin coating, in agreement with the compositional results indicating 7.6–9.1% lignin retention (Figure 2a, Section 3.1). This retained lignin layer is important for LCNF functional properties, including enhanced hydrophobicity, UV-blocking capacity, and antioxidant activity [63]. Minor differences in localized fibril entanglement among fractions are typical for mechanically isolated nanocellulose and do not indicate substantive differences in intrinsic fibril morphology [78].

Quantitative fibril diameter analysis was performed using ImageJ software on both FE-SEM and TEM images. Because the accurate measurement of individual fibrils within densely entangled networks is inherently challenging—a limitation widely noted in nanocellulose morphological characterization [61,77]—measurements were confined to regions where single fibrils could be clearly distinguished from the background and adjacent fibers. At least 50 well-defined nanofibrils were measured per sample to ensure statistical robustness. The mean diameters obtained from the combined FE-SEM and TEM analysis were 26.16 nm for LCNF-F45, 24.62 nm for LCNF-F100, and 25.84 nm for LCNF-F200 (Table 4). These values fall within a narrow range (24.62–26.16 nm), with a coefficient of variation below 6%, indicating that the initial particle size fractionation of the raw SCB feedstock had a negligible effect on the final nanofibril dimensions after sequential pretreatment and mechanical nanofibrillation.

The observed fibril diameter range (24.62–26.16 nm) is consistent with previously reported values for LCNF produced via mild pretreatment strategies [30,79,80]. Ferrer et al. (2012) reported fibril diameters of 25–40 nm for LCNF isolated from eucalyptus kraft pulp using enzymatic pretreatment [80], while Liu et al. (2019) obtained diameters of 28–35 nm from bamboo using an acidic deep eutectic solvent treatment [30]. In contrast, extensively delignified cellulose nanofibrils prepared by TEMPO-mediated oxidation commonly exhibit smaller diameters of 10–20 nm [28,79], reflecting more complete lignin removal and a reduced effective fibril cross-sectional. The moderately larger diameters observed here are attributed to the preserved lignin coating on fibril surfaces (7.6–9.1%), which increases the apparent fibril width while imparting functional properties that are absent in fully delignified nanocellulose [61,63]. This diameter regime is advantageous for applications requiring a balance mechanical reinforcement and functional surface chemistry, such as UV-protective coatings and antioxidant-active composites [17,66].

Beyond morphological uniformity, all LCNF suspensions exhibited highly negative zeta potential (Table 4), indicating excellent colloidal stability in aqueous media. Zeta potential values ranged from −86. 3 ± 0.92 mV (LCNF-F45) to −95.0 ± 1.03 mV (LCNF-F200), far exceeding the commonly used stability criterion (|ζ| > 30 mV) [81]. These results indicate that LCNF produced via sequential THT–DES pretreatment possess superior dispersibility without requiring additional chemical surface functionalization. Comparable values (−80 to −100 mV) have been reported for mechanically isolated nanocellulose [76], consistent with the role of mechanical shear in exposing charged surface functionalities.

The high surface charge is attributed to ionizable functional groups associated with retained lignin—particularly phenolic hydroxyl groups (pKa ~10)—as well as carboxyl moieties introduced during DES pretreatment [63]. These groups can deprotonate at neutral pH, stabilizing LCNF dispersions through electrostatic repulsion [63]. The slight increase in the magnitude of the zeta potential for larger initial particle sizes suggests minor variations in surface chemistry; however, particle size fractionation did not meaningfully affect the overall colloidal stability.

Overall, the combination of uniform nanofibril dimensions (24.62–26.16 nm), preserved lignin content (7.6–9.1%; Figure 2a), and high colloidal stability (|ζ| > 86 mV) demonstrates that sequential THT–DES pretreatment produces chemically and morphologically consistent LCNF across the fine particle size range (≤200 μm). Achieving this consistency without particle size-specific optimization of pretreatment conditions underscores the robustness of the sequential strategy and its suitability for producing high-performance LCNF with predictable properties for applications requiring hydrophobicity, UV protection, antioxidant functionality, and stable aqueous dispersion [22,24].

## 4. Conclusions

This study demonstrates the successful valorization of industrially relevant sub-millimeter SCB fractions into LCNF using an environmentally benign, sequential THT–DES pretreatment strategy. Three critical knowledge gaps in SCB-based LCNF research were systematically addressed: (i) comprehensive characterization of fine and ultra-fine fractions (≤45, 45–100, and 100–200 μm) that are naturally generated during industrial grinding and, yet, have been overlooked in prior DES-based pretreatment studies, which predominantly relied on commercially processed pulps or coarse particles (>200 μm) without systematic size fractionation; (ii) development of a sequential pretreatment approach explicitly designed for lignin retention (7.6–9.1%), in contrast to conventional single-step DES delignification that yields lignin-free substrates; and (iii) experimental validation that all evaluated fine fractions—including ultra-fines (≤45 μm), which fall below the particle size range typically targeted in NREL compositional analysis workflows (knife-milled to >180 μm) and are often discarded in biomass pretreatment studies due to concerns about excessive degradation and reduced solid recovery—can serve as viable LCNF precursors, enabling the complete valorization of industrial grinding output.

The sequential THT–DES pretreatment achieved efficient lignocellulosic fractionation while preserving cellulose structural integrity. THT (190 °C, 15 min) removed 65–67% hemicellulose via autohydrolysis, followed by DES treatment (ChCl:urea 1:2, 130 °C, 2 h) that resulted in hemicellulose removal of >70%, reduced lignin to 7.6–9.1%, and enriched cellulose to 74.0–77.5%. Multi-technique characterization (FT-IR, TGA, and XRD) consistently confirmed stepwise removal of non-cellulosic components, preservation of native cellulose I structure, and progressive crystallinity enhancement from 46.49–52.66% after THT to 56.69–57.19% after DES. Importantly, although THT-induced changes retained particle size dependence (*CrI* variation of 6.17% percentage points), the subsequent DES stage effectively eliminated these differences, yielding remarkably uniform crystallinity (<0.50% percentage points variation), thermal stability (peak temperature difference <5 °C), and chemical composition regardless of initial particle size. This particle size-independent outcome enables rational utilization of the full fine particle spectrum produced during industrial grinding, including ultra-fines that have traditionally been avoided in biomass pretreatment research.

Mechanical nanofibrillation (700 bar, five passes) produced LCNF with consistent properties across all fractions, including uniform fibril diameters (24.62–26.16 nm, <6% variation), visible lignin coating on fibril surfaces, and exceptional colloidal stability (zeta potential: −86.3 to −95.0 mV). The moderately larger diameters relative to fully delignified CNF (10–20 nm) are attributable to preserved lignin (7.6–9.1%), which confers functional attributes such as hydrophobicity, UV-blocking capacity, and antioxidant activity. Demonstrating the viability of ultra-fine fractions (≤45 μm) as LCNF precursors transforms these previously problematic byproducts of industrial grinding into valuable feedstocks, supporting the complete valorization of SCB grinding outputs and advancing circular bioeconomy objectives. Relative to prior SCB-based LCNF routes that require harsh chemicals (e.g., soda-oxygen pulping, toxic NaClO reagents, or corrosive acids) or high energy input (e.g., steam explosion), the sequential THT–DES strategy offers the following: (1) environmentally benign processing using bio-based and potentially recyclable solvents under mild conditions; (2) effective lignin preservation to enable functional LCNF; and (3) practical applicability across the full industrial particle size distribution without specialized preprocessing or size exclusion.

### Limitations and Future Perspectives

Several limitations should be acknowledged to enable a more comprehensive assessment of industrial scalability. First, although energy consumption, environmental toxicity, and costs are recognized barriers to CNF commercialization, quantitative techno-economic analysis (TEA) and life cycle assessment (LCA) were not conducted in this study. Future work should evaluate capital and operating costs, energy balances, environmental impacts, and market-competitive LCNF production costs while accounting for regional SCB availability. Second, pretreatment severity and mechanical nanofibrillation energy demand across the full industrial particle size distribution require systematic optimization. Third, direct links between LCNF structure (e.g., lignin content, fibril diameter, and crystallinity) and quantitative end-use performance in specific applications (composites, barrier films, UV protection, and antioxidant delivery) should be established for scale-up challenges—including reactor design, DES handling, and continuous nanofibrillation—and require further investigation to support industrial implementation.

Future research will prioritize the following: (1) comprehensive TEA and LCA comparing THT–DES–LCNF platform with conventional CNF routes and alternative SCB valorization pathways; (2) quantitative assessment of DES recovery, purification, and multi-cycle recycling performance; (3) systematic optimization across the complete industrial particle size distribution to minimize energy consumption and maximize LCNF yield and quality; (4) development of structure–property relationships linking LCNF characteristics to its application in relevant performance metrics; and (5) pilot-scale demonstration integrated with sugar-mill operations to validate technical, economic, and environmental feasibility. Collectively, these efforts will advance the THT–DES–LCNF platform from laboratory proof-of-concept toward commercially viable biorefinery solutions, enabling the conversion of abundant agricultural residues into high-value nanomaterials for sustainable development in sugar-producing regions worldwide.

## Figures and Tables

**Table 1:** Comparison of particle size treatments in DES-based lignocellulosic biomass pretreatment studies.

Reference	Biomass	Particle Size	Pretreatment	DES Type	Key Outcome
[46]	Birch pulp	Not reported	DES pretreatment followed by microfluidization	ChCl:urea (1:2)	90% yield; 2–5 nm CNF; *CrI* 66%; DP preserved
[47]	Poplar/Miscanthus	40~60 mesh (0.25~0.425 mm)	p-TsOH/ChCl DES pretreatment followed by NaOH post-treatment	ChCl:p-TsOH	*CrI* 68% (PL), 68.1% (MC); >90% lignin removal; 100% cellulose conversion
[48]	Bleached birch pulp	183–211 μm after DES	DES pretreatment followed by microfluidization	Betaine HCl:Glycerol	72.5% yield; 17–20 nm CNF; *CrI* 67.7–74.4%; 80–110 MPa film
[49]	Raw ramie fibers	~1 cm cut fibers	Acidic DES (ChCl:oxalic acid) pretreatment followed by ball milling	ChCl:oxalic acid (1:1)	73% recovery; 90.31% glucan; 14.29 nm CNF; 98.071 MPa film
[50]	Rice straw	2~10 mm cutting	Single-step DES	lactic acid: betaine and lactic acid:choline chloride	Extract >90% purity lignin;
[51]	SCB	Not reported	DES treatment followed by enzymatic hydrolysis	ChCl:Gly, ChCl:Urea	>80% lignin removal; 50~80% cellulose recovery

Note: ChCl = Choline chloride; p-TsOH = p-Toluenesulfonic acid; *CrI* = Crystallinity index; DP = Degree of polymerization; CNF = Cellulose nanofibrils; PL = Poplar; MC = Miscanthus.

**Table 2:** Comparison of LCNF production methods from lignocellulosic biomass: pretreatment strategies and lignin retention.

Reference	Biomass	Process Summary	Lignin Content	Main Results
[27]	SCB	Soda–oxygen pulping →disk refining →ultrasonication	~25% →4.46–16.11%	2–5 nm LCNF; improved thermal stability; lower temp than conventional
[28]	SCB	Organosolv pretreatment →TEMPO oxidation →ultrasonication /microfluidization	15~24% →5–7.5%	600–800 nm (length) LCNF; harsh NaClO oxidation
[29]	SCB	Steam explosion →alkaline delignification →enzymatic hydrolysis →high-pressure homogenization	~25% →4.8~11.6%	10~40 nm LCNF; micro/nanofibrils; high energy (steam); moderate lignin retention
[55]	Phragmites australis (reed)	Mild alkaline (NaOH) treatment →ball milling →ultrasonication	16% →7.3~16.1%	5~10 nm LCNF; ~87% LCNF yield; native-like lignin structure preserved
[30]	Date palm waste	Hydrothermal treatment →maleic acid (MA) treatment →high-pressure homogenization	28% →17~26%	High yield (>70%); lignin preservation; MA-assisted treatment
[55]	Phragmites australis (reed)	Mild alkaline (NaOH) treatment →ball milling →ultrasonication	16% →7.3~16.1%	5~10 nm LCNF; ~87% LCNF yield; native-like lignin structure preserved

Note: LCNF = Lignin-containing cellulose nanofibrils; TEMPO = 2,2,6,6-Tetramethylpiperidine-1-oxyl; NaClO = Sodium hypochlorite; MA = Maleic acid; THT = Thermal hydrolysis treatment; DES = Deep eutectic solvent; ChCl = Choline chloride.

**Table 3:** Crystallinity index of raw SCB and samples after sequential THT-DES pretreatment.

Sample	Crystallinity Index (%)
Raw SCB	49.83
THT-F45	46.49
THT-F100	48.31
THT-F200	52.66
DES-F45	57.06
DES-F100	56.69
DES-F200	57.19

**Table 4:** Morphological characteristics and colloidal properties of LCNF samples.

	Fibril Diameter (nm) ^a^	Zeta Potential (mV) ^b^
LCNF-F45	26.16	−86.3 ± 0.92
LCNF-F100	24.62	−92.5 ± 1.01
LCNF-F200	25.84	−95.0 ± 1.03

^a^ Determined from TEM and FE-SEM image analysis (*n* = 50 measurements per sample). ^b^ Measured by DLS at 0.1 wt% in deionized water at 25 °C.

## Data Availability

The original contributions presented in this study are included in the article. Further inquiries can be directed to the corresponding authors.

## References

[1] Pandey A., Soccol C.R., Nigam P., Soccol V.T. (2000). Biotechnological potential of agro-industrial residues. I: Sugarcane bagasse. Bioresour. Technol..

[2] Mandal A., Chakrabarty D. (2011). Isolation of nanocellulose from waste sugarcane bagasse (SCB) and its characterization. Carbohydr. Polym..

[3] Hofsetz K., Silva M.A. (2012). Brazilian sugarcane bagasse: Energy and non-energy consumption. Biomass Bioenergy.

[4] Mahmud M.A., Anannya F.R. (2021). Sugarcane bagasse-A source of cellulosic fiber for diverse applications. Heliyon.

[5] Jacobsen S.E., Wyman C.E. (2002). Xylose monomer and oligomer yields for uncatalyzed hydrolysis of sugarcane bagasse hemicellulose at varying solids concentration. Ind. Eng. Chem. Res..

[6] Karp S.G., Woiciechowski A.L., Soccol V.T., Soccol C.R. (2013). Pretreatment strategies for delignification of sugarcane bagasse: A review. Braz. Arch. Biol. Technol..

[7] Bezerra T.L., Ragauskas A.J. (2016). A review of sugarcane bagasse for second-generation bioethanol and biopower production. Biofuels Bioprod. Biorefin..

[8] Hiranobe C.T., Gomes A.S., Paiva F.F., Tolosa G.R., Paim L.L., Dognani G., Cabrera F.C. (2024). Sugarcane bagasse: Challenges and opportunities for waste recycling. Clean Technol..

[9] Matsueda Y., Antunes E. (2024). A review of current technologies for the sustainable valorisation of sugarcane bagasse. J. Environ. Chem. Eng..

[10] Culaba A.B., Mayol A.P., San Juan J.L.G., Vinoya C.L., Concepcion R.S., Bandala A.A., Chang J.S. (2022). Smart sustainable biorefineries for lignocellulosic biomass. Bioresour. Technol..

[11] Meng X., Pu Y., Li M., Ragauskas A.J. (2020). A biomass pretreatment using cellulose-derived solvent Cyrene. Green Chem..

[12] Gupta G.K., Shukla P. (2020). Lignocellulosic biomass for the synthesis of nanocellulose and its eco-friendly advanced applications. Front. Chem..

[13] Yu S., Sun J., Shi Y., Wang Q., Wu J., Liu J. (2021). Nanocellulose from various biomass wastes: Its preparation and potential usages towards the high value-added products. Environ. Sci. Ecotechnol..

[14] Santucci B.S., Bras J., Belgacem M.N., da Silva Curvelo A.A., Pimenta M.T.B. (2016). Evaluation of the effects of chemical composition and refining treatments on the properties of nanofibrillated cellulose films from sugarcane bagasse. Ind. Crops Prod..

[15] Yang M., Zhang X., Guan S., Dou Y., Gao X. (2020). Preparation of lignin containing cellulose nanofibers and its application in PVA nanocomposite films. Int. J. Biol. Macromol..

[16] Wu W., Zhu P., Luo L., Lin H., Tao Y., Ruan L., Qing Q. (2024). p-Toluenesulfonic acid enhanced neutral deep eutectic solvent pretreatment of soybean straw for efficient lignin removal and enzymatic hydrolysis. Bioresour. Technol..

[17] Sun Y., Zhang H., Li Q., Vardhanabhuti B., Wan C. (2022). High lignin-containing nanocelluloses prepared via TEMPO-mediated oxidation and polyethylenimine functionalization for antioxidant and antibacterial applications. RSC Adv..

[18] Berglund L.A., Peijs T. (2010). Cellulose biocomposites—From bulk moldings to nanostructured systems. MRS Bull..

[19] Liu X., Hao M., Chen L., Yang G., Liang C., He Y., Ma P., Pan M., Xie W., Jian R. (2021). Lignin-containing cellulose nanomaterials: Preparation and applications. Green Chem..

[20] Eichhorn S.J., Dufresne A., Aranguren M., Marcovich N.E., Capadona J.R., Rowan S.J., Weder C., Thielemans W., Roman M., Renneckar S. (2010). Current international research into cellulose nanofibres and nanocomposites. J. Mater. Sci..

[21] Nishino T., Matsuda I., Hirao K. (2004). All-cellulose composite. Macromolecules.

[22] Chen L., Zhu J.Y., Baez C., Kitin P., Elder T. (2024). Lignin-containing cellulose nanomaterials: Preparation, properties, and applications. Rare Met..

[23] Chen X.Y., Gao X.J., Wu H.Y., Liu Y.L., Yang X.F., Sun R.C. (2024). Lignin-reinforced PVDF electrolyte for dendrite-free quasi-solid-state Li metal battery. Rare Met..

[24] Huang Z., Han D., Yi G., Lin W., Lin X., Sun Y., Wang H. (2025). High-performance and multifunctional lignin-derived polyurethane elastomers for robotic flexible protective layers. Adv. Funct. Mater..

[25] Henriksson M., Henriksson G., Berglund L.A., Lindström T. (2007). An environmentally friendly method for enzyme-assisted preparation of microfibrillated cellulose (MFC) nanofibers. Eur. Polym. J..

[26] Huang C., Lin W., Lai C., Li X., Jin Y., Yong Q. (2019). Coupling the post-extraction process to remove residual lignin and alter the recalcitrant structures for improving the enzymatic digestibility of acid-pretreated bamboo residues. Bioresour. Technol..

[27] Yao L., Yang H., Yoo C.G., Meng X., Pu Y., Hao N., Ragauskas A.J. (2022). Facile preparation of lignin-containing cellulose nanofibrils from sugarcane bagasse by mild soda-oxygen pulping. Cellulose.

[28] Scopel E., Mota T.R., Nunes V.M., Mazzaro I., Lacerda T.M., Pinto P.C. (2025). Residual lignin affects production and properties of TEMPO-oxidized cellulose nanofibrils from partially delignified sugarcane bagasse. Cellulose.

[29] Fontes A.M., Iamamoto Y., Correa A.C., Bilatto S., Brienzo M., Cavali M., Moraes M.L., Mattoso L.H.C. (2021). Micro/nanostructured lignonanocellulose obtained from steam-exploded sugarcane bagasse. Cellulose.

[30] Liu X., Li Y., Ewulonu C.M., Ralph J., Xu F., Zhang Q., Wu M., Huang Y. (2019). Mild alkaline pretreatment for isolation of native-like lignin and lignin-containing cellulose nanofibers (LCNF) from crop waste. ACS Sustain. Chem. Eng..

[31] Najahi A., Hamrouni R., Samaali I., Hentati O., Boufi S. (2023). High-lignin-containing cellulose nanofibrils from date palm waste produced by hydrothermal treatment in the presence of maleic acid. Biomacromolecules.

[32] Abolore R.S., Jaiswal S., Jaiswal A.K. (2024). Green and sustainable pretreatment methods for cellulose extraction from lignocellulosic biomass and its applications: A review. Carbohydr. Polym. Technol. Appl..

[33] Mankar A.R., Pandey A., Modak A., Pant K.K. (2021). Pretreatment of lignocellulosic biomass: A review on recent advances. Bioresour. Technol..

[34] Yang Y., Zhang M., Zhao J., Wang D. (2023). Effects of particle size on biomass pretreatment and hydrolysis performances in bioethanol conversion. Biomass Convers. Biorefin..

[35] Heidarian P., Behzad T., Karimi K. (2016). Isolation and characterization of bagasse cellulose nanofibrils by optimized sulfur-free chemical delignification. Wood Sci. Technol..

[36] Andrade M.F., Colodette J.L. (2014). Dissolving pulp production from sugar cane bagasse. Ind. Crops Prod..

[37] Francisco M., Van Den Bruinhorst A., Kroon M.C. (2012). New natural and renewable low transition temperature mixtures (LTTMs): Screening as solvents for lignocellulosic biomass processing. Green Chem..

[38] Liu W., Du H., Liu K., Liu H., Xie H., Si C., Zhang X. (2021). Sustainable preparation of cellulose nanofibrils via choline chloride-citric acid deep eutectic solvent pretreatment combined with high-pressure homogenization. Carbohydr. Polym..

[39] Li M., Cao S., Meng X., Studer M., Wyman C.E., Ragauskas A.J., Pu Y. (2017). The effect of liquid hot water pretreatment on the chemical–structural alteration and the reduced recalcitrance in poplar. Biotechnol. Biofuels.

[40] Anastas P.T., Warner J.C. (2000). Green Chemistry: Theory and Practice.

[41] Zhu J.Y., Pan X.J. (2010). Woody biomass pretreatment for cellulosic ethanol production: Technology and energy consumption evaluation. Bioresour. Technol..

[42] Auxenfans T., Crônier D., Chabbert B., Paës G. (2017). Understanding the structural and chemical changes of plant biomass following steam explosion pretreatment. Biotechnol. Biofuels.

[43] Sluiter A., Hames B., Ruiz R., Scarlata C., Sluiter J., Templeton D., Crocker D. (2008). Determination of structural carbohydrates and lignin in biomass. Lab. Anal. Proced..

[44] Kumar R., Hu F., Sannigrahi P., Jung S., Ragauskas A.J., Wyman C.E. (2013). Carbohydrate derived-pseudo-lignin can retard cellulose biological conversion. Biotechnol. Bioeng..

[45] Sluiter J.B., Ruiz R.O., Scarlata C.J., Sluiter A.D., Templeton D.W. (2010). Compositional analysis of lignocellulosic feedstocks. 1. Review and description of methods. J. Agric. Food Chem..

[46] Sun J.X., Sun X.F., Zhao H., Sun R.C. (2004). Isolation and characterization of cellulose from sugarcane bagasse. Polym. Degrad. Stab..

[47] Sirviö J.A., Visanko M., Liimatainen H. (2015). Deep eutectic solvent system based on choline chloride-urea as a pre-treatment for nanofibrillation of wood cellulose. Green Chem..

[48] Zhou Z., Lei F., Li P., Jiang J. (2021). Recyclable deep eutectic solvent coupling sodium hydroxide post-treatment for boosting woody and herbaceous biomass conversion at mild condition. Bioresour. Technol..

[49] Hong S., Yuan Y., Yang Q., Zhu P., Lian H. (2020). Enhancement of the nanofibrillation of birch cellulose pretreated with natural deep eutectic solvent. Ind. Crops Prod..

[50] Yu W., Wang C., Yi Y., Zhou W., Wang H., Yang Y., Zeng Z., Guo Z. (2021). Direct pretreatment of raw ramie fibers using an acidic deep eutectic solvent to produce cellulose nanofibrils in high purity. Cellulose.

[51] Kumar A.K., Parikh B.S., Pravakar M. (2016). Natural deep eutectic solvent mediated pretreatment of rice straw: Bioanalytical characterization of lignin extract and enzymatic hydrolysis of pretreated biomass residue. Environ. Sci. Pollut. Res..

[52] Varilla-Mazaba C.A., Morales-Contreras B.E., Rodríguez-González F., Wicker L., Rosas-Flores W., Rodríguez-Núñez J.R., Morales-Castro J. (2022). Choline chloride-based deep eutectic solvents as biocompatible extractants to recover hemicellulosic sugars and phenolic compounds from sugarcane bagasse. ACS Omega.

[53] Yang H., Yan R., Chen H., Zheng C., Lee D.H., Liang D.T. (2006). In-depth investigation of biomass pyrolysis based on three major components: Hemicellulose, cellulose and lignin. Energy Fuels.

[54] Qing Q., Yang B., Wyman C.E. (2010). Impact of surfactants on pretreatment of corn stover. Bioresour. Technol..

[55] Da Silva A.S.A., Inoue H., Endo T., Yano S., Bon E.P. (2010). Milling pretreatment of sugarcane bagasse and straw for enzymatic hydrolysis and ethanol fermentation. Bioresour. Technol..

[56] (2024). Standard Specification for Woven Wire Test Sieve Cloth and Test Sieves.

[57] (2013). Test Sieves—Technical Requirements and Testing—Part 2: Test Sieves of Perforated Metal Plate.

[58] Yanak S., Buyukkileci A.O. (2024). Delignification of corncob by choline chloride-urea deep eutectic solvent for enzymatic production of xylooligosaccharides. Ind. Crops Prod..

[59] Segal L., Creely J.J., Martin A.E., Conrad C.M. (1959). An empirical method for estimating the degree of crystallinity of native cellulose using the X-ray diffractometer. Text. Res. J..

[60] Schneider C.A., Rasband W.S., Eliceiri K.W. (2012). NIH Image to ImageJ: 25 years of image analysis. Nat. Methods.

[61] Herrera M., Thitiwutthisakul K., Yang X., Rujitanaroj P.-O., Rojas R., Berglund L. (2018). Preparation and evaluation of high-lignin content cellulose nanofibrils from eucalyptus pulp. Cellulose.

[62] Nitsos C.K., Matis K.A., Triantafyllidis K.S. (2013). Optimization of hydrothermal pretreatment of lignocellulosic biomass in the bioethanol production process. ChemSusChem.

[63] Nair S.S., Yan N. (2015). Effect of high residual lignin on the thermal stability of nanofibrils and its enhanced mechanical performance in aqueous environments. Cellulose.

[64] Sulaeman A.P., Gao Y., Dugmore T., Remón J., Matharu A.S. (2021). From unavoidable food waste to advanced biomaterials: Microfibrilated lignocellulose production by microwave-assisted hydrothermal treatment of cassava peel and almond hull. Cellulose.

[65] Wang Q.Q., Zhu J.Y., Reiner R.S., Verrill S.P., Baxa U., McNeil S.E. (2012). Approaching zero cellulose loss in cellulose nanocrystal (CNC) production: Recovery and characterization of cellulosic solid residues (CSR) and CNC. Cellulose.

[66] Espinosa E., Bascón-Villegas I., Rosal A., Pérez-Rodríguez F., Chinga-Carrasco G., Rodríguez A. (2019). PVA/(ligno)nanocellulose biocomposite films. Effect of residual lignin content on structural, mechanical, barrier and antioxidant properties. Int. J. Biol. Macromol..

[67] Klemm D., Kramer F., Moritz S., Lindström T., Ankerfors M., Gray D., Dorris A. (2011). Nanocelluloses: A new family of nature-based materials. Angew. Chem. Int. Ed..

[68] Stefanidis S.D., Kalogiannis K.G., Iliopoulou E.F., Lappas A.A., Pilavachi P.A. (2014). A study of lignocellulosic biomass pyrolysis via the pyrolysis of cellulose, hemicellulose and lignin. J. Anal. Appl. Pyrolysis.

[69] Yang H., Yan R., Chen H., Lee D.H., Zheng C. (2007). Characteristics of hemicellulose, cellulose and lignin pyrolysis. Fuel.

[70] Poletto M., Zattera A.J., Santana R.M.C. (2012). Structural differences between wood species: Evidence from chemical composition, FTIR spectroscopy, and thermogravimetric analysis. J. Appl. Polym. Sci..

[71] Baraka F., Erdocia X., Velazco-Cabral I., Labidi J. (2024). Impact of deep eutectic solvent pre-treatment on the extraction of cellulose nanofibers. Cellulose.

[72] Bhattacharjee S. (2016). DLS and zeta potential–What they are and what they are not?. J. Control. Release.

[73] Sukkathanyawat H., Bampenrat A., Jarunglumlert T., Prommuak C. (2022). Modification of waste sugarcane bagasse fly ash for CO_2_ capture application. Mater. Renew. Sustain. Energy.

[74] French A.D. (2014). Idealized powder diffraction patterns for cellulose polymorphs. Cellulose.

[75] Pereira S.C., Maehara L., Machado C.M.M., Farinas C.S. (2016). Physical-chemical- morphological characterization of the whole sugarcane lignocellulosic biomass used for 2G ethanol production by spectroscopy and microscopy techniques. Renew. Energy.

[76] Nitsos C.K., Choli-Papadopoulou T., Matis K.A., Triantafyllidis K.S. (2016). Optimization of hydrothermal pretreatment of hardwood and softwood lignocellulosic residues for selective hemicellulose recovery and improved cellulose enzymatic hydrolysis. ACS Sustain. Chem. Eng..

[77] Nagarajan K.J., Ramanujam N.R., Sanjay M.R., Siengchin S., Ranjith R., Rajeshkumar G., Ramesh M. (2021). A comprehensive review on cellulose nanocrystals and cellulose nanofibers: Pretreatment, preparation, and characterization. Polym. Compos..

[78] Nair S.S., Zhu J.Y., Deng Y., Ragauskas A.J. (2014). Characterization of cellulose nanofibrillation by micro grinding. J. Nanopart. Res..

[79] Jiang F., Hsieh Y.L. (2013). Chemically and mechanically isolated nanocellulose and their self-assembled structures. Carbohydr. Polym..

[80] Ferrer A., Quintana E., Filpponen I., Solala I., Vidal T., Rodríguez A., Laine J., Rojas O.J. (2012). Effect of residual lignin and heteropolysaccharides in nanofibrillar cellulose and nanopaper from wood fibers. Cellulose.

[81] Saito T., Kimura S., Nishiyama Y., Isogai A. (2007). Cellulose nanofibers prepared by TEMPO-mediated oxidation of native cellulose. Biomacromolecules.

